# BMC Hematology reviewer acknowledgement 2015

**DOI:** 10.1186/s12878-016-0044-x

**Published:** 2016-02-22

**Authors:** Nawsheen Boodhun

**Affiliations:** BioMed Central, Floor 6, 236 Gray’s Inn Road, London, WC1X 8HB UK

## Abstract

The editors of *BMC Hematology* would like to thank all our reviewers who have contributed to the journal in Volume 15 (2015).

**Omar Aljitawi**

USA

**David Archer**

USA

**Michael Auerbach**

USA

**Sudipto Bari**

Singapore

**John Belisle**

USA

**Lealem Bimerew**

Ethiopia

**Kyle Bradley**

USA

**Michael Briones**

USA

**Jorg Bungert**

USA

**Keyne Charlot**

France

**Seth Corey**

USA

**Wim De Kort**

Netherlands

**Anthony Dear**

Australia

**Neville Dossabhoy**

USA

**Lisa Duffett**

Canada

**Kerstin Edlefsen**

USA

**Sameer Elsayed**

Canada

**Richard Francis**

USA

**Luca Galli**

Italy

**Esteban Gandara**

Canada

**Eugenio Gaudio**

Switzerland

**Natalia Giltiay**

USA

**Nicola Glaser**

Germany

**Thelma T Goncalez**

USA

**Isabelle Gouin-Thibault**

France

**Mark Heaney**

USA

**Tohru Ikuta**

USA

**Ronald Jaffe**

USA

**Philippe Joly**

France

**Karen Kalinyak**

USA

**Julie Kanter**

USA

**Prashant Kapoor**

USA

**Micaela La Regina**

Italy

**Chong Lei**

China

**Jane Leopold**

USA

**Brea Lipe**

USA

**Chen Liu**

USA

**Pipat Luksamijarulkul**

Thailand

**Federico Lussana**

Italy

**Tomer Mark**

USA

**Luca Masotti**

Italy

**Michele Merli**

Italy

**Jonathan Metts**

USA

**Erin Meyer**

USA

**Jaime Modiano**

USA

**Daya Moodley**

South Africa

**Sangita Mukhopadhyay**

Iran

**Nicola Mumoli**

Italy

**Sergei Nekhai**

USA

**Susie Nilsson**

Australia

**Vikki Nolan**

USA

**Ntobeko Ntusi**

Zimbabwe

**Maureen Okam**

USA

**Michael Owusu**

Ghana

**Amy Pai**

USA

**Roger Pearse**

USA

**Susan Perrine**

USA

**Vincent Pialoux**

France

**Fulvio Pomero**

Italy

**Valerie Proulle**

France

**Vinod Pullarkat**

USA

**Savita Rangarajan**

UK

**Linda Resar**

USA

**Davide Rossi**

Italy

**Elisa Rumi**

Italy

**Santosh Saraf**

USA

**Alan Schechter**

USA

**Marie P Schneider**

Switzerland

**Jatin Shah**

USA

**Salimah Shariff**

Canada

**Shamee Shastry**

India

**Anurag Singh**

USA

**Georg Stüssi**

Switzerland

**Paola Tacchetti**

Italy

**Joseph Telfair**

USA

**Minh-Ha Tran**

USA

**Tugba Tumer**

Turkey

**Nienke van Rein**

Netherlands

**Angel Vargas-Ruiz**

Mexico

**Cheryl Vigen**

USA

**José Vitale**

Italy

**Andrew Wilber**

USA

**Alauldeen Zubair**

Iraq

